# Effects of the open lung concept following ARDSnet ventilation in patients with early ARDS

**DOI:** 10.1186/s12871-016-0206-1

**Published:** 2016-07-20

**Authors:** Vivian Rotman, Alysson Roncally Carvalho, Rosana Souza Rodrigues, Denise Machado Medeiros, Eduardo Costa Pinto, Fernando Augusto Bozza, Carlos Roberto Ribeiro Carvalho

**Affiliations:** 1 Internal Medicine Department, Federal University of Rio de Janeiro, Rua Reseda, 23/401, Rio de Janeiro, 22471-230 Brazil; 2 Laboratory of Respiration Physiology, Instituto de Biofísica Carlos Chagas Filho, Federal University of Rio de Janeiro, Rio de Janeiro, Brazil; 3 Department of Radiology, Federal University of Rio de Janeiro, Rio de Janeiro, Brazil; 4 D’Or Institute for Research and Education (IDOR), Rio de Janeiro, Brazil; 5 Instituto de Pesquisa Clínica Evandro Chagas (IPEC), Oswaldo Cruz Foundation, Rio de Janeiro, Brazil; 6 ICU, Copa D’Or Hospital, Rio de Janeiro, Brazil; 7 Respiratory ICU, InCor-Hospital das Clinicas, University of Sao Paulo, Sao Paulo, Brazil

**Keywords:** Lung -adult respiratory distress syndrome, Measurement techniques – tomography, Ventilation – effects

## Abstract

**Background:**

Ventilation with low tidal volume (V_T_) is well recognized as a protective approach to patients with acute respiratory distress syndrome (ARDS), but the optimal level of positive end-expiratory pressure (PEEP) remains uncertain. This study aims to evaluate two protective ventilatory strategies sequentially applied in patients with early ARDS.

**Methods:**

In this prospective cohort study, fifteen patients were ventilated during 24 h with positive end-expiratory pressure (PEEP) adjusted according to the ARDSnet low-PEEP table (ARDSnet-24 h). During the next 24 h, nine patients with PaO2/FIO2 ratio below 350 mmHg were ventilated with PEEP titrated according to the Open Lung Concept protocol (ARDSnet + OLC). In the other six patients, regardless of their PaO2/FIO2 ratio, the ARDSnet remained for a further 24 h (ARDSnet-48 h). Ventilatory variables, arterial blood-gas and cytokine were obtained at baseline, 24 and 48 h. Additionally, whole-lung-computed tomography was acquired at 24 and 48 h.

**Results:**

A sustained improvement in PaO2/FIO2 ratio (*P* = 0.008) with a decrease in collapsed regions (*P* = 0.008) was observed in the ARDSnet + OLC group compared with the ARDSnet-24 h group. A reduction in IL-6 in plasma (*P* < 0.02) was observed throughout the protocol in the ARDSnet + OLC group. Compared with the ARDSnet-48 h group, the ARDSnet + OLC presented smaller amounts of collapsed areas (*P* = 0.018) without significant differences in hyperinflated regions and in driving and plateau pressures.

**Conclusions:**

In this set of patients with early ARDS, mechanical ventilation with an individually tailored PEEP sustained improved pulmonary function with better aeration, without significant increase in hyperinflated areas”.

**Trial registration:**

Brazilian Clinical Trials Registry (ReBec). RBR-5zm9pr. 04th November 2015

## Background

Ventilatory strategies employing high tidal volumes (V_T_) have been shown to induce lung injury and are associated with increased mortality in patients with Acute Respiratory Distress Syndrome (ARDS) [1, 2]. Although the reduction in V_T_ is well recognized as a protective approach [3, 4], the optimal level of positive end-expiratory pressure (PEEP) remains uncertain [5–7].

Large trials comparing high and low levels of PEEP with low V_T_ failed to show differences in mortality [8–10], although an improvement in oxygenation was generally observed in the higher PEEP groups [9, 10]. This improvement, however, may be transitory and/or have no impact on mortality. The potential benefits of high levels of PEEP seem to be dependent upon the severity of pulmonary injury [6, 7] and may reduce mortality in patients with persistent and severe hypoxemia, preventing lung collapse and further hypoxia [6, 11].

Although it has already been shown that recruitment maneuvers associated with PEEP titration determine improvement in lung compliance and oxygenation, and reduction in cytokines levels, these analyses were mainly done in a transversal way. In the research that made a prolonged analysis (until 40 h), there was no tomographic evaluation. A prolonged longitudinal conjoint analysis of lung aeration by computed tomography (CT) scan and cytokines is lacking [12, 13].

In the present study, we evaluated the effects on pulmonary function, aeration and inflammatory response of two protective ventilatory strategies with low and high levels of PEEP individually titrated and sequentially applied in patients with early-established ARDS. Our analysis focused on the effects of each ventilatory strategy regarding oxygenation, lung mechanics, pulmonary aeration and inflammation after 24 h of mechanical ventilation. Our hypothesis is that individually titrated PEEP levels add benefits to protective ventilatory strategies.

## Methods

### Patient enrollment

The present study was conducted at the Hospital Copa D’Or, a tertiary care hospital located in Rio de Janeiro, Brazil. The Hospital Institutional Review Board (IRB) approved the study protocol (number: HCB027/05) and written informed consent (including the permission to publish indirect identifiers) was obtained from all patients or next-of-kin relatives. Fifteen patients with clinical ARDS diagnosis [14] with PaO_2_/FIO_2_ ratios lower than 300 mmHg after 30 min of mechanical ventilation with a PEEP of 5 cmH_2_O, an FIO_2_ of 1.0 and a V_T_ ranging from 8 to 10 mL/kg of ideal body weight (IBW), along with hemodynamically stable parameters (mean arterial pressures higher than 65 mmHg and arterial lactates lower than 3 mmoL/L) over the preceding 6 h [15], were enrolled in the study.

Patients were excluded if more than 48 h had elapsed since their diagnosis of ARDS, or if any of the following clinical conditions were identified: pneumothorax, pneumomediastinum, bronchopleural fistula, subpleural blebs, increased intracranial pressure, a body weight greater than 140 kg, pregnancy, or a coexistent disease with an expected 6-month mortality risk exceeding 50 %.

### Experimental protocol

**Fig. 1:**
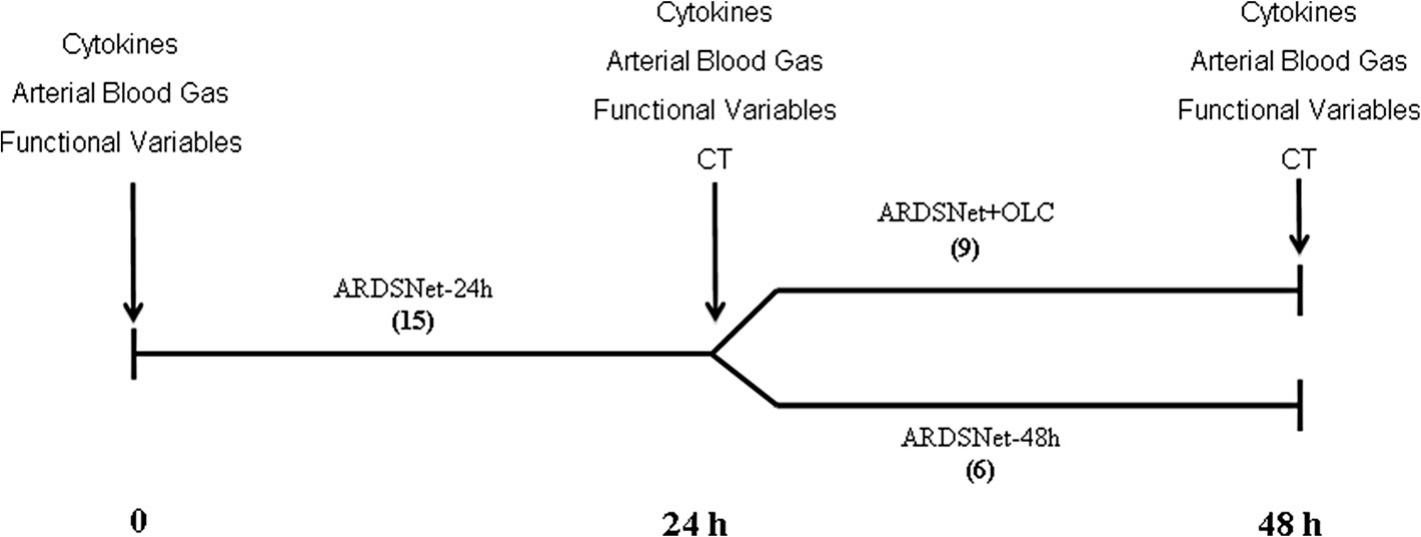
Schematic representation of the time-line interventions used during the protocol. At baseline, samples for cytokine and arterial blood gas measurements, as well as functional data, were acquired. After the first 24 h of mechanical ventilation according to the ARDSnetwork protocol, a new set of functional measurements was performed, including a whole-lung CT-scan exam. Patients were subsequently divided into two groups (ARDSnet-48 h or ARDSnet + OLC). At the end of the protocol (a total of 48 h of observation), measurements were repeated, and a new CT-scan was acquired

Figure 1 summarizes the experimental protocol. Briefly, all patients were kept sedated and lying in a supine position while being ventilated (SERVOi, MAQUET, Sweden) with the bed elevated at 30°. Baseline data, including ventilatory variables and arterial blood samples for gas and cytokine analyses were obtained just after patient inclusion. Thereafter, all patients were ventilated according to the ARDSnetwork low-PEEP table [2], with a V_T_ of 6 mL/kg (ranging from 4 to 8 mL/kg), and plateau pressure maintained below 30 cmH_2_O over the next 24 h. After this period, respiratory variables and arterial blood samples were collected, and patients were transported to the Radiology Department for CT-scan imaging, in SERVOi, without ventilator disconnection (ARDSnet-24 h). After 24 h of ventilation according to ARDSnet, the first nine patients with the PaO_2_/FIO_2_ ratio below 350 mmHg were ventilated according to the Open Lung Concept (OLC) for the next 24 h (ARDSnet + OLC). The next six patients, regardless of their PaO_2_/FIO_2_ ratios, served as controls and remained on the ARDSnet-low PEEP table protocol for an additional 24 h (ARDSnet-48 h). At the end of this period, arterial blood samples, respiratory variables and a new whole-lung CT-scan were acquired.

### Open lung concept protocol

#### Recruitment maneuver (RM)

In the OLC protocol, an RM was performed in Pressure Controlled Ventilation (PCV): driving pressure of 20 cmH_2_O; inspiratory time of 3 s; inspiratory to expiratory ratio (I:E) of 1:1; FIO_2_ of 1.0 and PEEP sequentially increased to 25 and 30 cmH_2_O; two minutes per step. Thereafter, driving pressure was adjusted to keep a V_T_ of 6 mL/kg (IBW) and respiratory rate (RR) was increased to 15 bpm with I:E ratio of 1:2–1:3 [14].

### PEEP titration

Immediately after the RM, PEEP was adjusted to 17 cmH_2_O while keeping all other parameters constant for at least 20 min [16]. After this period, if the PaO_2_/FIO_2_ ratio was lower than 350 mmHg [16], the last step of the RM (driving pressure of 20 cmH_2_O and PEEP of 30 cmH_2_O) was repeated. Thereafter the driving pressure was adjusted to provide a V_T_ of 6 mL/kg and the PEEP was adjusted to 19 cmH_2_O. Twenty minutes afterwards, a new blood sample was collected and if the PaO_2_/FIO_2_ ratio remained below 350 mmHg, the last step of the RM was repeated and after driving pressure adjustment PEEP was set at 21 cmH_2_O. Twenty minutes later, if the PaO_2_/FIO_2_ ratio was still lower than 350 mmHg, the PEEP associated with the highest PaO_2_/FIO_2_ ratio was selected.

During the OLC protocol, the driving pressure was continuously adjusted to keep Pplat below 30 cmH_2_O and the pH higher than 7.2, with the respiratory rate ranging from 15 to 20/min. No further RMs were performed throughout the OLC protocol.

### CT acquisition and analysis

Whole-lung helical CT-scan images were acquired during an expiratory breath-hold maneuver (6 s) at 24 and 48 h of the protocol. During CT acquisitions, patients were sedated, paralyzed and ventilated with an FIO_2_ of 1.0 according to the respective ventilatory strategy.

CT images were acquired on one of three scanners: a Light Speed QX/i, a Light Speed Pro 16, or a Light Speed VCT (GE Medical Systems, Milwaukee, WI). CT scans were obtained sequentially using the following parameters: 0.8 s (Light Speed QX/i) or 0.5 s (Light Speed Pro 16 and Light Speed VCT) axial time rotations, 1.25-mm section reconstructions, 5-mm intervals, 120 kVp, 150–250 mA (Light Speed QX/i) or mA adjustment (Light Speed Pro 16 and Light Speed VCT), and a 512 × 512 pixel matrix.

Image analyses were performed with a purpose-built routine written in MatLab (Mathworks) by one of the authors (ARC). The region of interest (ROI) was manually selected in each slice by taking the inner contour of the thorax but excluding the chest wall, mediastinum, pleural effusions and regions presenting partial volume effects. For each ROI, the total volume of hyperaerated (−1,000 to −900 Hounsfield units-HU), normally (−900 to −500 HU), poorly (−500 to −100 HU) and non-aerated (−100 to +100 HU) regions were computed and color mapped [17]. Additionally, the total mass of each compartment was calculated [16, 18]. From the total lung volume, 3D reconstructions were created by masking the lung boundary as well as each aeration compartment.

### Cytokine analysis

Arterial blood samples, collected at Baseline, 24 and 48 h during the protocol, were centrifuged and the plasma stored at −70 °C for subsequent cytokine analysis.

Interleukins 6 and 8 (IL-6 and −8) were measured using commercially available ELISA kits in accordance with the manufacturer’s instructions (R&D Systems, Minneapolis, EUA).

### Statistical analysis

Data are presented as median, first and third quartiles. Differences were considered significant if *P* < 0.05. The temporal effects related to changes in aeration, ventilatory variables, and cytokine analyses in each group were compared with the Wilcoxon sign rank test for paired samples. Comparisons between groups (ARDSnet-48 h and ARDSnet + OLC) were performed with the Mann–Whitney test using the SPSS statistical package (Version 16.0, SPSS, Inc.).

## Results

Baseline parameters are presented in Table 1. The majority of the patients included had pneumonia, and none had trauma. Both groups were comparable regarding age, Apache II, SOFA, time of mechanical ventilation, respiratory system compliance and PaO_2_/FIO_2_ ratio (Table 1). According to the Berlin Definition [14] ARDS was classified as mild in 10 patients (6 in ARDSnet + OLC and 4 in ARDSnet-48 h), moderate in 4 (2 in each group) and severe in one patient (in ARDSnet + OLC group). Outcomes are presented in Table 2.

**Table 1:** Baseline characteristics of the patients

Group	Age	Gender	Apache II	Basal SOFA	Underlying disease	Time on MV at baseline (hours)	Crs (mLcmH_2_O)	PaO_2_/FlO_2_
ARDSnet + OLC	71	F	23	7	Pneumonia	12	34	217
ARDSnet + OLC	76	M	17	7	Pneumonia	11	20	179
ARDSnet + OLC	63	M	16	8	Pneumonia	24	33	250
ARDSnet + OLC	73	M	18	8	Pneumonia	22	41	246
ARDSnet + OLC	62	F	11	7	Pneumonia/Acute Pancreatitis	7	25	226
ARDSnet + OLC	31	M	25	5	Pneumonia	48	30	220
ARDSnet + OLC	50	F	22	7	Pneumonia	7	58	229
ARDSnet + OLC	79	M	20	5	Pneumonia	24	32	78
ARDSnet + OLC	46	F	32	11	Multiple transfusions	24	25	171
Median (1QR)	63 (48–75)		20 (17–24)	5(4–7)		22 (9–24)	32 (25–37)	220 (175–237)
ARDSnet-48 h	24	M	17	8	Pneumonia	6	45	201
ARDSnet-48 h	66	M	10	4	Urinary sepsis	48	51	278
ARDSnet-48 h	88	F	19	6	Pneumonia	24	40	125
ARDSnet-48 h	41	M	16	4	Pneumonia	48	53	279
ARDSnet-48 h	88	M	19	7	Pneumonia	13	35	266
ARDSnet-48 h	66	M	15	7	Pneumonia	16	28	144
Median (1QR)	66 (37–88)		17 (14–19)	7 (5–8)		20 (11–48)	43 (33–52)	233 (139–278)
Overal Median (1QR)	63 (48–75)		20 (17–24)	7 (6–8)		15 (8–28)	32 (25–37)	220 (175–237)

*M* male, *F* female, *Apache*, Acute Physiology and Chronic Health Evaluation II score, *SOFA* Sequential Organ Failure Assessment, *MV* mechanical ventilation, *Crs* respiratory system compliance, *PaO*_*2*_,/*FIO*_*2*_ arterial pressure of oxygen/inspiratory fraction of oxygen ratio

**Table 2:** Patients outcomes

Group	Barotrauma	Liberation of MV	ICU death	Hospital death	Day of death	Days of ICU	Days of hospitalization
ARDSNet + OLC	No	Yes	No	No		10	18
ARDSNet + OLC	No	Yes	No	No		36	53
ARDSNet + OLC	No	No	Yes	Yes	15	16	16
ARDSNet + OLC	No	Yes	No	No		28	75
ARDSNet + OLC	No	Yes	No	No		19	195
ARDSNet + OLC	No	Yes	No	No		40	59
ARDSNet + OLC	Yes	No	Yes	Yes	106	107	107
ARDSNet + OLC	No	No	Yes	Yes	17	31	31
ARDSNet + OLC	No	Yes	Yes	Yes	13	14	14
ARDSNet	No	Yes	No	No		4	10
ARDSNet	No	Yes	No	No		12	14
ARDSNet	No	No	Yes	Yes	0	2	3
ARDSNet	No	Yes	No	No		15	38
ARDSNet	No	Yes	No	No		13	30
ARDSNet	No	No	Yes	Yes	14	17	17
						28 (15–38)	53 (17–91)

Day of death means time interval (d) between protocol completion and patient death

*MV* mechanical ventilation, *ICU* intensive care unit

### Gas exchange, hemodynamic and respiratory variables

Respiratory variables are presented in Table 3. A significant increase in PaCO_2_ (*P* = 0.002) and a reduction in pH (*P* = 0.017) were observed after the first 24 h of ARDSnet strategy compared with Baseline. The institution of the OLC led to a significant and sustained improvement in the PaO_2_/FIO_2_ ratio (*P* = 0.008) with increased respiratory system compliance (*P* = 0.04) compared with Baseline. No significant difference was observed in PaCO_2_ (*P* = 0.26), driving or plateau pressures (*P* = 0.17 and 0.05, respectively). No significant improvement in oxygenation was observed in the ARDSnet-48 h group. The use of vasopressors and MAP levels were comparable among groups, although a lower fluid balance was observed in the OLC group (*P* = 0.04).

**Table 3:** Respiratory variables

	ARDSnet + OLC (*n* = 9)			ARDSnet-48 h (*n* = 6)		
	Baseline	ARDSnet-24 h	ARDSnet + OLC	Baseline	ARDSnet-24 h	ARDSnet-48 h
Pplat (cmH_2_O)	27 (23 – 27)	22 (20 – 26)	30 (24–31)	18 (15 – 25)	18 (15 – 23)	20 (17–24)
PEEP (cmH_2_O)	5 (5 – 5)	8 (8 – 11)	17 ^a^ ^b^ (17–19)	5 (5 – 5)	10 (5 – 11)	10 (7–11)
Driving Pressure (cmH_2_O)	14 (12 – 19)	12 (10 – 18)	12 (5–13)	13 (9 – 18)	10 (7 – 14)	11 (9.25 - 14)
V_T_ (mL/kg)	10 (10 – 10)	8^a^ (7 – 8)	7^a^ (6–8)	10 (10 – 10)	7^a^ (6 – 8)	7^a^ (6–8)
VE (L/min)	9 (8 – 11)	8 (8 – 10)	7 (6–9)	9 (8 – 11)	7 (6 – 8)	7 (6–8)
Crs (mL/cmH_2_O)	32 (25 – 37)	42 (23 – 48)	46^a^ (32–91)	43 (33 – 52)	48 (33 – 68)	40 (34–53)
PaO_2_/FIO_2_ (mmHg)	220 (175 – 238)	195 (184 – 246)	270^a^ ^b, c^ (220–363)	233 (139 – 278)	162 (132 – 282)	177 (100–283)
SaO_2_	100 (96 – 100)	98 (96 – 98)	99 (98 – 99)	100 (99 – 100)	98^a^ (94 – 99)	99^a^ (97 – 100)
PaCO_2_ (mmHg)	41 (28 – 44)	44^a^ (41 – 51)	49^a^ (42–58)	37 (34 – 39)	47^a^ (40 – 55)	43 (39–55)
pH	7.4 (7.3 – 7.4)	7.3 (7.3 – 7.4)	7.3 (7.3 - 7.4)	7.3 (7.3 – 7.5)	7.3 (7.2 – 7.4)	7.4 (7.2 - 7.4)

*Pplat* plateau pressure of the respiratory system, *Driving Pressure* inspiratory pressure above PEEP in Pressure Controlled Ventilation Mode

*V*_*T*_ tidal volume in mL/kg of predicted body weight, *VE* minute ventilation, *PEEP* positive end-expiratory pressure, *PaO*_*2*_/*FIO*_*2*_ arterial pressure of oxygen/inspiratory fraction of oxygen ratio, *Crs* respiratory system compliance, *PaCO*_*2*_ arterial pressure of carbon dioxide, *SaO*_*2*_ arterial blood saturation. Data is presented as median, first and third quartiles

^a^Significant differences compared to Baseline

^b^Significant differences compared to ARDSnet-24 h

^c^Significant differences compared to ARDSnet-48 h

No hemodynamic instability was observed during recruitment. During PEEP titration, three patients did not reach a PaO_2_/FIO_2_ ratio higher than 350 mmHg, even at the highest PEEP level employed (21 cmH_2_O). The PEEP was then adjusted according to the maximal oxygenation level reached at 19 cmH_2_O in 2 cases and 17 cmH_2_O in the other case.

### Quantitative and qualitative CT analysis

Morphological CT-scan data are shown in Table 4. A significant increase in the fraction of normally aerated areas in parallel with a decrease in collapsed regions (*P* = 0.008) was observed after 24 h with OLC compared with the first 24 h with the ARDSnet protocol. No significant changes in aeration were observed in the ARDSnet-48 h group when compared with the first 24 h with ARDSnet. Comparing the OLC group with the ARDSnet-48 h group, a higher fraction of normally aerated (*P* = 0.015), with a lower fraction of collapsed regions (*P* = 0.018), was observed. No significant difference in hyperinflated regions was observed (*P* = 0.179) among groups.

**Table 4:** Quantitative CT analysis

	ARDSnet + OLC (*n* = 9)		ARDSnet-48h (*n* = 6)	
	ARDSnet-24h	ARDSnet + OLC	ARDSnet-24h	ARDSnet + OLC
Hyper Mass (%)	0 (0–1)	1 0–2)	0 (0–0.5)	0 (0–0.5)
Norm Mass (%)	20 (13–22.5)	38^a^ (31–42)	26.5 (15–35)	25 (20–39)
Poorly-aerated Mass (%)	25 (18–32)	25 (18–42)	17 (14–21)	16.5 (14–20)
Non-aerated Mass (%)	58 (51–60)	30^a^ (23–49)	58 (47–68)	60.5 (43–65)
Hyper Volume (%)	2 (0–7)	5 (1–14)	0 (0–4)	0 (0–2)
Norm Volume (%)	41 (31–47)	61^a^ (53–63)	52 (36–63)	52.5 (41–66)
Poorly aerated Volume (%)	21 (15–30)	14 (11–29)	14 (10–16)	12.5 (11–16)
Non-aerated Volume (%)	37 (31–41)	13^a^ (11–23)	33 (24–48)	35 (20–44)

Hyper Mass, fraction of non-air content in the hyperinflated compartment related to the total lung mass; Norm Mass, fraction of non-air content in the normally aerated compartment related to the total lung mass

*Poorly*-*aerated Mass* fraction of non-air content in the poorly aerated compartment related to the total lung mass, *Non*-*aerated Mass* fraction of non-air content in the non aerated compartment related to the total lung mass. Data is presented as median, first and third quartiles

^a^Significant differences compared to ARDSnet-24h

**Fig. 2:**
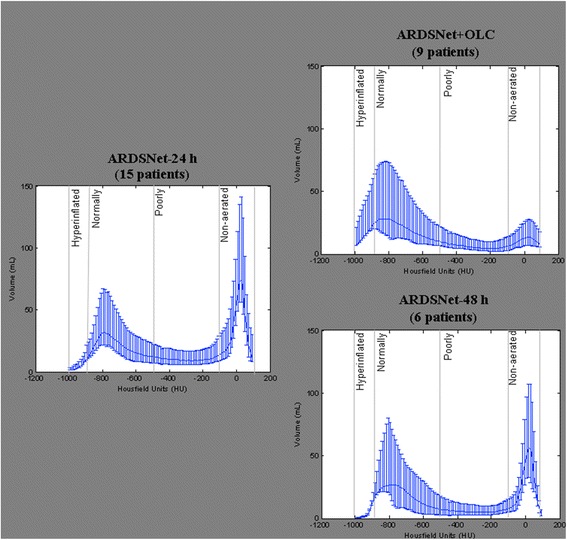
Histograms of the distribution of aeration (in Hounsfield Units (HU)) in patients ventilated with the ARDSnetwork protocol during the first 24 h (left hand panel) followed by ventilation using either the OLC protocol (right hand upper panel) or the ARDSnetwork protocol (right hand lower panel) for the next 24 h. The histograms are presented as the median, first and third quartiles

The distribution of voxels per HU in each protocol is presented in Fig. 2. The OLC (Fig. 2, upper right panel) exhibited a unimodal distribution with a peak in the normally aerated compartment, whereas the histograms of the ARDSnet group exhibited a bimodal pattern with two peaks, one in the normally-aerated and the other in the non-aerated compartment after 24 (Fig. 2, left panel) and 48 h (Fig. 2, lower right panel) of mechanical ventilation.

**Fig. 3:**
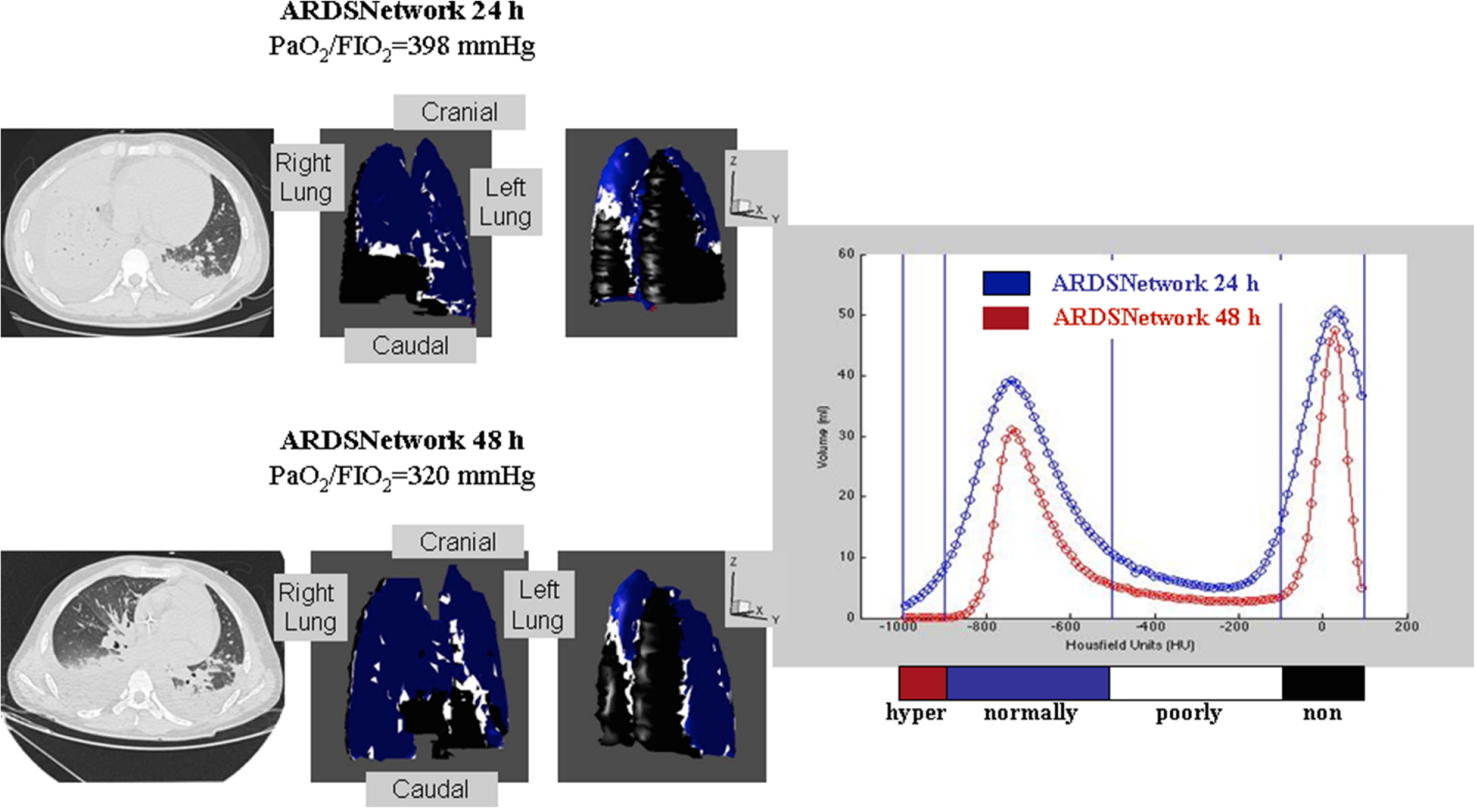
Representative 3D reconstructions with the respective histograms of voxels distribution of a patient ventilated in ARDSnet-48 h group. A predominance of collapsed areas can be observed (coloured in black) in the dorsal regions during ARDSnet-24 h (left upper panels). The bimodal pattern persisted after 48 h of mechanical ventilation with the ARDSnet protocol (right panel)

**Fig. 4:**
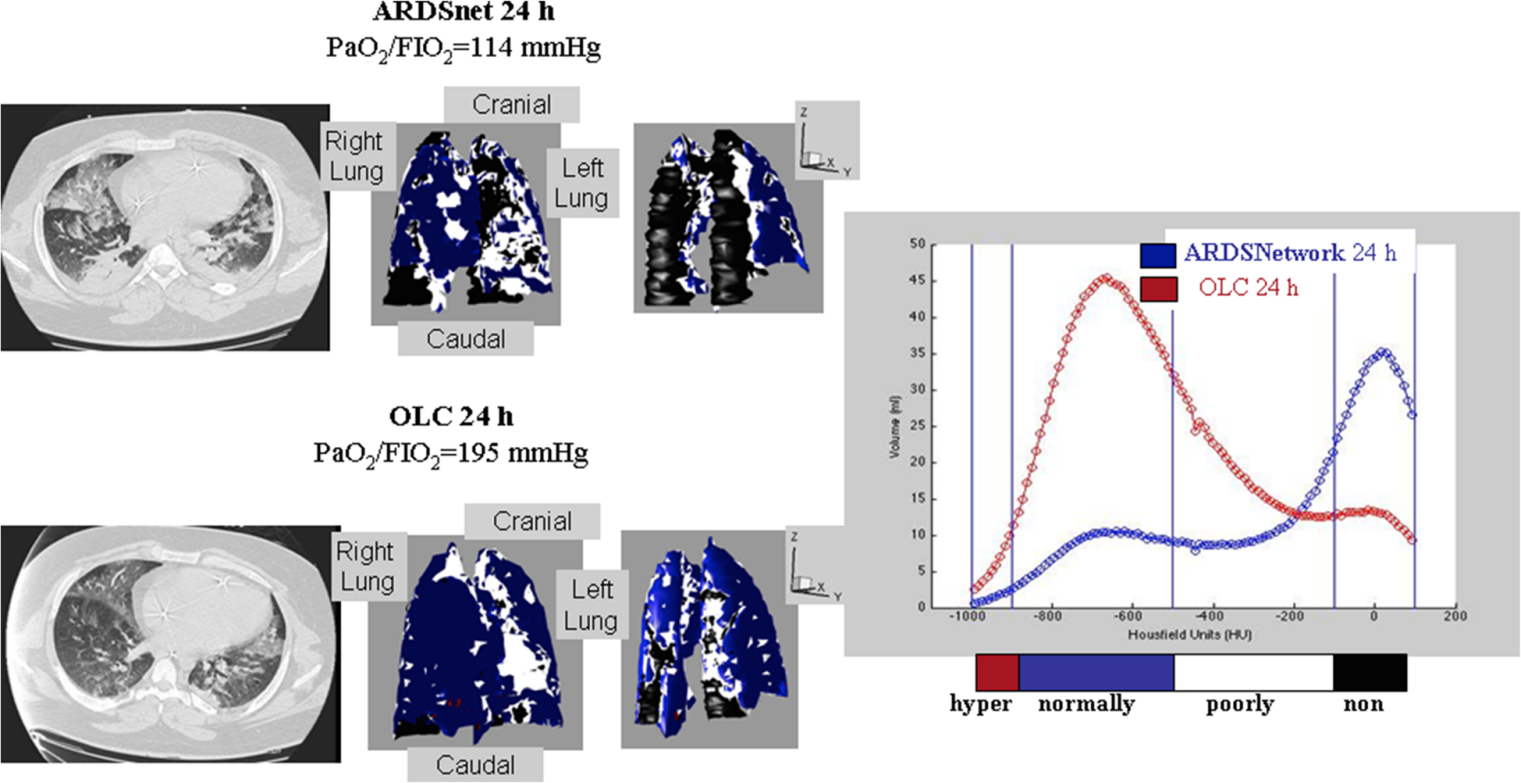
Representative 3D reconstructions with the respective histograms of voxels distribution of a patients ventilated in ARDSnet + OLC group. A predominance of collapsed areas can be observed (coloured in black) in the dorsal regions during ARDSnet-24 h (left upper panels), whereas the OLC resulted in a shift from non- to normally aerated regions (right panel)

Representative 3D reconstructions with the respective histograms of voxels distribution of two patients are shown in Figs. 3 and 4. A predominance of collapsed areas can be observed (coloured in black) in the dorsal regions during ARDSnet-24 h (Figs. 3 and 4 upper left panels). Furthermore, the bimodal pattern persisted after 48 h of mechanical ventilation with the ARDSnet protocol (Fig. 3, right panel), whereas the OLC resulted in a shift from non to normally aerated regions (Fig. 4, right panel).

### Cytokine analysis

**Fig. 5:**
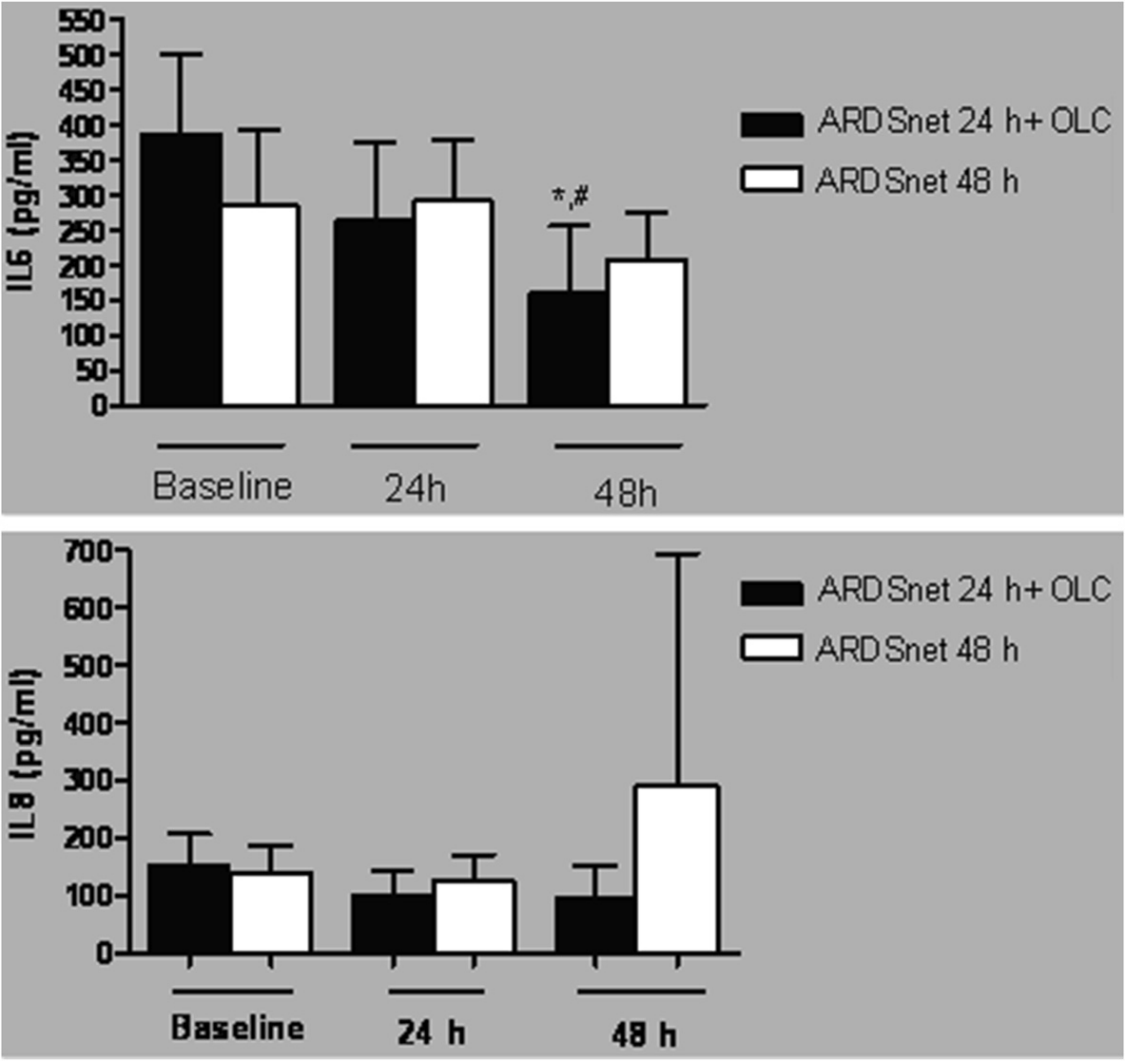
Plasma concentrations of IL-6 and IL-8 during the protocol. Plasma concentrations of IL-6 decreased after 24 h of mechanical ventilation with OLC compared with both Baseline (*P* = 0.02) and 24 h of ARDSnetwork (*P* = 0,005). No significant difference was observed in IL-8 among groups

Plasma concentrations of IL-6 decreased after 24 h of mechanical ventilation with OLC compared with Baseline (*P* = 0.02) and ARDSnet 24 h (*P* = 0,005 - Fig. 5). No significant difference was observed in IL-8 plasma concentration measurements.

## Discussion

The main findings of the present study were: 1) a sustained improvement in oxygenation with a reduction in non and poorly aerated areas was observed after 24 h of mechanical ventilation with the OLC; 2) no significant increase in driving or plateau pressures, hyperinflated areas or cytokine levels occurred with OLC.

The hazardous effects of ventilation with high volumes are well known [15, 19]. Since the ARDSnet trial [2], protective ventilation with low tidal volume and limited plateau pressure has been the standard of care for patients with ALI/ARDS [3, 20]. Although the concept of atelectrauma is also well established, strategies for its prevention (high levels of PEEP and RMs) are still under debate [21]. Some studies [8–10] failed to show a reduction in mortality with the adoption of high levels of PEEP; however, in two of these [9, 10], high PEEP led to a reduction in the incidence of severe hypoxemia that required rescue therapy. In this context, this study was designed to explore the pathophysiological effects of OLC in comparison with a more conservative protective ventilatory strategy, aiming to evaluate the prolonged longitudinal conjoint effects on pulmonary aeration and cytokines.

Regarding oxygenation and respiratory system mechanical properties, there was a sustained improvement in oxygenation after 24 h of ventilation with the OLC, as well as an increase in respiratory system compliance in agreement with previous results [10]. The benefits of RMs and high PEEPs may depend on lung recruitability, which may be highly variable [11]. It has been recently shown that higher levels of PEEP improved survival only in patients with severe ARDS [22]. The majority of our patients had just mild ARDS [14]. Despite this, we found a significant reduction in non and poorly aerated areas, without significant increase in hyperinflated areas, plateau and driving pressures. Thus, we believe that our favorable results are a consequence of the adoption of effective RMs. Indeed, the few clinical studies that used maximal recruitment in combination with open-lung PEEP resulted in significant and sustained improvements in oxygenation, as well as in lung mechanics [9, 10], and in other studies, there was even a reduction in mortality [5, 23]. There is a close interdependence between recruitment and PEEP effects. Without an effective RM, the actual open-lung PEEP is difficult to assess [24, 25].

In the current study, maximal pressure levels higher than 40 cmH_2_O were applied during the RMs, which appears to be necessary to open the more dependent areas of the lung [16]. The use of a PEEP that keeps PaO_2_/FIO_2_ ratio higher than 350 mmH_2_O seems to result in less than five percent of the collapsed areas [16].

In our study, RMs with high pressures were not associated with deleterious hemodynamic effects. This was associated with the inclusion of patients with early ALI/ARDS who have less stiff chest walls and higher recruitment potentials [11, 19].

As previously reported [26], RM with high airway pressures have the potential to induce overdistension leading to an increased production of inflammatory cytokines from different cells in the alveolus and pulmonary vasculature. Nevertheless, cyclic closing and overdistension, induced by lung units with different time-constants, and ventilation with inadequate PEEP levels are also associated with higher levels of cytokines in the alveolar space and blood [27, 28]. In the present study, plasma cytokine concentrations were measured at baseline, 24 and 48 h after patient inclusion. Despite the use of higher and more sustained pressure inflation in our RM protocol compared to other trials, we found significantly lower plasma levels of IL-6 in the OLC group when compared with the Baseline and 24 h time points of the ARDSnet. These results are in accordance with CT analysis, where OLC was associated with reduction in non and poorly aerated areas without significant hyperinflation.

The sequential approach adopted in the OLC group suggests the possibility that the progressive fall in plasma IL-6 levels may have resulted as a consequence of an improvement in clinical status. To evaluate this possibility, we analyzed a second group, which was ventilated for 48 h in the ARDSnet protocol (ARDSnet-48 h), but we did not find significant changes in cytokine levels (Fig. 5).

The study has limitations. First, it had a small sample size as it was designed to evaluate physiological variables. Second, it was done during 48 h, so inferences regarding clinical outcomes and security cannot be made after this period. Also, as it was a 48 h study, we could not assure that spontaneous breaths were not present all the time, and if so, probably it would be present more often during ventilation with higher PEEP levels and could interfere with the results in the OLC group. It is also important to emphasize that comparisons between the final results of the two groups (ARDSnet + OLC and ARDSnet-48 h) should be done with caution as patients with worse PaO_2_/FIO_2_ ratios were allocated in the ARDSnet + OLC group, maybe supporting the differences in the results favoring this group.

In summary, our data suggest that the OLC provides an effective approach to reduce the fraction of collapsed tissue without significantly increasing the fraction of hyperinflated tissue and without increasing plasma cytokine levels. We believe that this strategy could provide a viable alternative treatment option for patients with ALI/ARDS. Further studies are warranted to confirm our results and to identify subgroups of patients that will benefit the most from this strategy.

## Conclusions

Mechanical ventilation based on an OLC, applying an individually tailored PEEP, can induce sustained improvement in pulmonary function, with better pulmonary aeration and less hyperinflated areas, without increment in cytokines.

## Abbreviations

Apache, acute physiology and chronic health evaluation II score; ARDS, early acute respiratory distress syndrome; ARDSnet, ARDS network; CT, computed tomography; HU, hounsfield units; I:E, inspiratory to expiratory ratio; IBW, ideal body weight; IL, interleukins; IRB, The hospital Institutional review board; MAP, mean arterial pressure ; OLC, open lung concept protocol; PaCO_2_, arterial pressure of carbon dioxide; PaO2/FIO2, arterial pressure of oxygen/inspiratory fraction of oxygen ratio; PCV, pressure controlled ventilation; PEEP, positive end-expiratory pressure; RM, Recruitment maneuver; ROI, region of interest; RR, respiratory rate; SOFA, sequential organ failure assessment; V_T_, tidal volume
